# The Selection of Reference Genes for Quantitative Real-Time PCR in the Ashidan Yak Mammary Gland During Lactation and Dry Period

**DOI:** 10.3390/ani9110943

**Published:** 2019-11-10

**Authors:** Xiaoyun Wu, Xuelan Zhou, Xuezhi Ding, Min Chu, Chunnian Liang, Jie Pei, Lin Xiong, Pengjia Bao, Xian Guo, Ping Yan

**Affiliations:** Key Laboratory of Yak Breeding Engineering, Lanzhou Institute of Husbandry and Pharmaceutical Sciences, Chinese Academy of Agricultural Sciences, Lanzhou 730050, China; wuxiaoyun@caas.cn (X.W.); zhouxl17@lzu.edu.cn (X.Z.); dingxuezhi@caas.cn (X.D.); chumin@caas.cn (M.C.); liangchunnian@caas.cn (C.L.); peijie@caas.cn (J.P.); xionglin@caas.cn (L.X.); baopengjia@caas.cn (P.B.)

**Keywords:** reference gene, RT-qPCR, mammary gland

## Abstract

**Simple Summary:**

The Ashidan yak is a new cultivated breed which has polled characteristics and a mild temperament. Improving milk yield is an important aspect of a breeding program for this breed. The mammary gland undergoes dramatic physiological and metabolic changes during the transition from lactation to dry periods, which involves the expression and regulation of a great number of genes. Quantification of gene expression levels by real-time quantitative polymerase chain reaction (RT-qPCR) is important to reveal the molecular mechanisms of mammary gland development and lactation. The accuracy of RT-qPCR is strongly influenced by the expression stability of reference genes, however, a systematic approach for selecting reference genes used for analyzing gene expression of the Ashidan yak has not been developed. In this study, we selected reference genes and analyzed their expression stability at different physiological stages (lactation and dry period). We found the hydroxymethylbilane synthase gene (*HMBS*) and the tyrosine 3-monooxygenase/tryptophan 5-monooxygenase activation protein, zeta polypeptide gene (*YWHAZ*) were the most stable genes of the mammary gland of the Ashidan yak. These results help to improve the accuracy of gene expression analysis and provide a basis for future functional studies of target gene expression in the mammary gland of the Ashidan yak.

**Abstract:**

Investigating the critical genes related to milk synthesis is essential for the improvement of the milk yield of the yak. Real-time quantitative polymerase chain reaction (RT-qPCR) is a reliable and widely used method to measure and evaluate gene expression levels. Selection of suitable reference genes is mandatory to acquire accurate normalization of gene expression results from RT-qPCR. To select the most stable reference genes for reliable normalization of mRNA expression by RT-qPCR in the mammary gland of the Ashidan yak, we selected 16 candidate reference genes and analyzed their expression stability at different physiological stages (lactation and dry period). The expression stability of the candidate reference genes was assessed using geNorm, NormFinder, BestKeeper, Delta Ct, and RefFinder methods. The results showed that the hydroxymethylbilane synthase gene (*HMBS*) and the tyrosine 3-monooxygenase/tryptophan 5-monooxygenase activation protein, zeta polypeptide gene (*YWHAZ*) were the most stable genes across all treatment samples. The reliability of selected reference genes was validated by normalizing relative expression of the lactation-related 60S ribosomal protein L35 gene (*RPL35*). The relative expression of *RPL35* varied considerably according to the different reference genes. This work provides valuable information to further promote research in the molecular mechanisms involved in lactation and mammary gland development and provides a foundation for the improvement of the milk yield and quality of the Ashidan yak.

## 1. Introduction

The yak (*Bos grunniens*) is one of the most crucial livestock species living on the Qinghai Tibetan Plateau, in areas of altitude ranging from 2500 m to 5500 m. It provides meat, milk, wool, transportation, and home heating for the indigenous human population [1]. Yak milk is considered a natural concentrated dairy product because of its higher nutritional value with a significant percentage of protein, essential minerals, and healthy polyunsaturated fatty acids, for example, conjugated linoleic acid and omega three fatty acids as compared with the milk of dairy cattle [2,3], however, the lower milk yield of yak (150 to 500 kg of fresh milk per lactation) severely limits the large-scale industrial production of yak milk [4]. The Ashidan yak is a newly cultivated breed which has the polled characteristics and mild temperament. In addition, it is easier to manage and feed in stalls compared to horned yak [5]. Nevertheless, milk production and quality traits of the Ashidan yak have been neglected in breeding programs. Therefore, improving the milk yield of the Ashidan yak meets the need for a dual-purpose breed in the yak industry.

The mammary gland is an essential organ for the synthesis and secretion of milk and undergoes dramatic physiological and metabolic changes during the transition from dry periods to lactation [6]. The dry period is critical for optimal milk production in the succeeding lactation. Manipulation of the transition from the dry period to lactation can directly affect mammary gland development and milk production [7,8]. To better understand factors regulating this transition, transcriptome studies have extensively explored the development process of the mammary gland and have found that many genes related to lactogenesis, milk secretion, and mammary gland development had differential expression in the mammary gland during the lactation and dry periods [9,10,11]. A quantitative examination of expression patterns of lactation-related genes can provide some insights into molecular regulatory mechanisms of metabolic and biological changes in the mammary gland.

Currently, real-time quantitative polymerase chain reaction (RT-qPCR) is one of the most used techniques for the evaluation of gene expression levels, owing to its conceptual and practical simplicity, together with its advantages of speed, sensitivity, specificity, reproducibility, high throughput, and a high degree of automation [12,13]. Reference gene selection for RT-qPCR data normalization is important to minimize the influence of RNA quality, reverse transcription, and primer efficiency [14]. Changes in reference gene stability have significant effects on the relative expressions of target genes. Ideally, reference genes should have consistent expression levels under various conditions, such as different organs or different developmental stages [15], however, in many cases, the expressions of reference genes has been found to vary under different conditions [16,17]. Therefore, it is critical to evaluate the stability of reference genes before normalization.

In order to identify the most appropriate reference genes and quantify the expression of the lactation-related genes in the mammary gland at different physiological conditions, 16 candidate reference genes were selected for evaluation, including β-actin (*ACTB*), β2 microglobulin (*B2M*), glyceraldehyde 3 phosphate dehydrogenase (*GAPDH*), mitochondrial ribosome-associated GTPase 1 (*MTG1*), hydroxymethylbilane synthase (*HMBS*), hypoxanthine guanine phosphoribosyl transferase 1 (*HPRT1*), mitochondrial ribosomal protein L39 (*MRPL39*), peptidylprolyl isomerase A (*PPIA*), protein phosphatase 1 regulatory subunit 11 (*PPP1R11*), ribosomal protein L13a (*RPL13A*), ribosomal protein S9 (*RPS9*), ribosomal protein S15 (*RPS15*), succinate dehydrogenase complex subunit A (*SDHA*), TATA-box binding protein (*TBP*), ubiquitously expressed transcript protein (*UXT*), and tyrosine 3-monooxygenase/tryptophan 5-monooxygenase activation protein, and zeta polypeptide (*YWHAZ*) [18,19,20,21]. The stabilities of these reference genes were assessed using geNorm, NormFinder, BestKeeper, Delta Ct, and RefFinder methods. The expression levels of the lactation related gene 60S ribosomal protein L35 (*RPL35*) as an objective gene were used to validate the selected reference genes.

## 2. Materials and Methods

### 2.1. Animals and Tissue Collection

All of the experimental protocols and procedures were performed according to the Animal Administration and Ethics Committee of the Lanzhou Institute of Husbandry and Pharmaceutical Sciences of CAAS (permit no. SYXK-2014-0002). Six multiparous, healthy and mastitis-free female yaks in their second parity, from different families, were selected for collecting mammary tissues. These yaks were kept under the same natural conditions with natural grazing and had free access to water with no supplementary feeding. Lactation yaks (*n* = 3) were suckling a calf (approximately 5 months of age) and not pregnant at the time of sample collection. The yaks at dry period (*n* = 3) were calved in the previous year with no pregnancy and no milking at the time of sample collection. The selected female yaks were slaughtered in September 2017, which is a time point close to the lactation peak (120 days postpartum). After slaughter, the rear mammary gland from each individual was removed within 30 min after slaughter. Mammary alveolar tissue was dissected from the middle of the upper one-third of the gland of a rear quarter of each yak, immediately frozen in liquid nitrogen, and stored at −80 °C until processed for total RNA extraction.

### 2.2. RNA Isolation and cDNA Synthesis

Total RNA from mammary tissues was extracted using Trizol reagent (Invitrogen, Carlsbad, CA, USA) following the manufacturer’s instructions. The integrity of total RNA was confirmed by inspection of 18S and 28S rRNA bands after 1.5% agarose gel electrophoresis. The RNA concentration and purity were assessed spectrophotometrically by NanoDrop2000 spectrophotometer (Thermo Fisher Scientific, Waltham, MA, USA). The purity of the total RNA was determined by the optical density (OD) ratio of OD_260_/OD_230_. The first strand complementary DNA (cDNA) was transcribed from 1 μg RNA using PrimeScriptTM RT reagent Kit with gDNA Eraser (TaKaRa, Dalian, China) according to the manufacturer’s instructions.

### 2.3. Selection of Candidate Reference Genes and Primer Design

A total of 16 stable reference genes were chosen for further evaluation of gene stability, according to previous studies. Primers for *TBP*, *ACTB*, *PPIA*, *HPRT1*, *GAPDH,* and *SDHA* were reported by Li et al. [22]. *B2M*, *MTG1*, *HMBS*, *MRPL39*, *PPP1R11*, *RPL13A*, *RPS9*, *RPS15*, *UXT,* and *YWHAZ* were designed based on the sequence obtained from NCBI using Primer Premier 5.0 software (Premier Biosoft International, Palo Alto, CA, USA). The size of the RT-qPCR products was between 79 and 190 bp.

### 2.4. Quantitative Real-Time PCR with SYBR Green

The RT-qPCR was carried out on a LightCycler 96 Real-Time PCR system (Roche Diagnostics, Mannheim, Germany) with a total volume of 20 μL per reaction. Each reaction mixture contained a 20 μL final volume containing 1 μL of first-strand cDNA, 10 µL of SYBR TB Green mix (TaKaRa, Dalian, China), 1 μL of forward primer (10 μM), 1 μL of reverse primer (10 μM), and 7 μL of ddH_2_O. The PCR program for amplification was as follows: 95 °C for 30 s, following by 45 cycles of 95 °C for 10 s, 60 °C for 10 s, and 72 °C for 20 s. To verify the specificity of PCR products, a melting curve analysis step was performed: 65 °C to 97 °C with a 0.5 °C increment and 2–5 s/step. Three replicates at the RT-qPCR step were used to ensure against a failed reaction. Standard curves of each candidate reference gene were constructed using a ten-fold dilution of cDNA from a RNA pool of known amounts of RNA from six yak mammary tissues. PCR amplification efficiency (E) was then determined according to the formula as follows:

$$E=(10^{\left(-1/\mathrm{slope}\right)}-1)\times 100.$$

### 2.5. Selection of Candidate Reference Genes and Primer Design

Quantification cycle (Cq) values for the 16 selected reference genes were imported into the spread sheet to evaluate the gene expression levels. The stability of the candidate genes was analyzed using four Excel-based statistical methods, geNorm [23], Norm Finder [24], BestKeeper [25], and Delta Ct [26]. A comprehensive web-based tool, RefFinder (http://150.216.56.64/referencegene.php), was used to generate the overall ranking of the candidate reference genes based on the geometric mean for the previously obtained ranks [27].

### 2.6. Validation of Reference Gene Stability

To validate the reference genes selected in our experimental system, the expression level of RPL35 was evaluated with RT-qPCR analysis. The expression levels of RPL35 in the mammary gland at different periods were normalized using the two most stable reference genes and the one least stable reference gene identified from this study. The relative mRNA expression data of *RPL35* gene was calculated using the 2^−ΔΔCt^ method. Statistical significance was analyzed using Student’s *t* test. Values with *p* < 0.05 were considered as statistically significant.

## 3. Results

### 3.1. Selection of Candidate Reference Genes and PCR Efficiency

In this study, 16 reference genes were selected based on previous literature and evaluated as candidate reference genes for RT-qPCR analyses. Details of gene symbol, NCBI accession number, primer pair sequences, and E values are displayed in Table 1. Primer specificity for each gene was validated by a melting curve analysis in qRT-PCR and a unique melting peak was observed in each case (Figure S1). The E values of all primer pairs were calculated by the LightCycler 96 system. Among the candidate reference genes, the E values ranged from 1.97 to 2.17, and R^2^ were not less than 0.99 (Table 2).

### 3.2. Expression Levels of the Candidate Reference Genes

**Figure 1:**
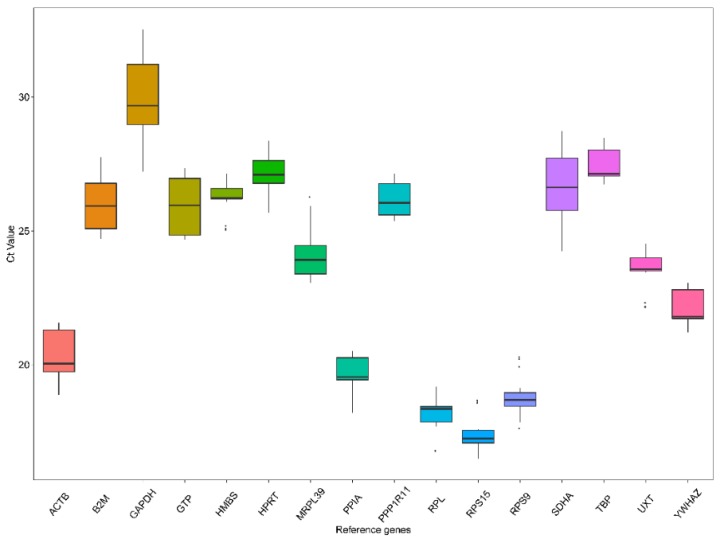
Cq values (expression levels) of 16 candidate reference genes in all tested samples. Each box indicates the 25th and 75th percentiles. Whisker caps correspond to the maximum and minimum values. A line within the boxes depicts the median.

The Cq values were determined to quantify expression levels of candidate reference genes in the samples by RT-qPCR. A lower Cq value means higher gene expression levels and vice versa. The Cq values of the 16 reference genes across all samples were displayed in a box and whiskers plot (Figure 1). The Cq values ranged from 16.49 to 32.53. The median Cq value was close to the mean Cq value for each of the reference genes. Among all the candidate reference genes, the *GAPDH* gene had the lowest expression level with Cq ranges of 29.94 ± 1.53, whereas the *RPS15* gene exhibited the highest expression with Cq value ranging between 17.41 ± 0.63.

### 3.3. Analysis of Gene Expression Stability of the Reference Genes

The expression stability of 16 candidate reference genes was determined using the geNorm, BestKeeper, NormFinder, and Delta Ct methods. The RefFinder method generates the comprehensive ranking and the geNorm method calculates the expression stability values (M) by comparing the average variation of each reference gene to all others. A lower M value indicates that a reference gene has higher stability. According to the geNorm method, all 16 genes had M values below the recommended cutoff value of 1.5, indicating that all reference genes possessed highly stable expression levels. The *HMBS* and *UXT* genes, with the lowest M values of 0.13, were the most stably expressed genes, whereas *GAPDH* was the least stable gene with the highest M value of 0.79. The NormFinder method ranks genes based on the average expression stability values (SVs). Genes with more stable expression have a lower SV. On the basis of the NormFinder results, *YWHAZ* and *TBP* were considered as the best reference genes with SVs of 0.22 and 0.24, while *GAPDH,* with an SV of 1.10, showed higher variation (Table 2). The BestKeeper method analyzes the expression stability of genes by determining which genes had the lowest standard deviation (SD). Similar to the geNorm method, *HMBS* (SD: 0.40) was highlighted as the most stably expressed gene. Conversely, *GAPDH* was the least stably expressed gene (SD: 1.21). Delta Ct is a comparative method that determines the most stable reference genes based on the means of the SD values. The ranking based on the Delta Ct method was very similar to NormFinder. *YWHAZ* (0.61) was selected as the most stable gene with the lowest SD value, and TBP (0.65) was the second most stable gene, whereas *GAPDH* (1.18) was the most variably expressed gene.

The RefFinder method integrates the four commonly used computational methods (geNorm, NormFinder, BestKeeper, and Delta Ct method) to avoid divergent ranking of the stable genes. The results showed that *HMBS* (2.51) and *YWHAZ* (2.63) were the most stable reference genes, and the least stable reference gene was *GAPDH* (16.00) (Table 2).

### 3.4. Best Reference Gene Number Identification

**Figure 2:**
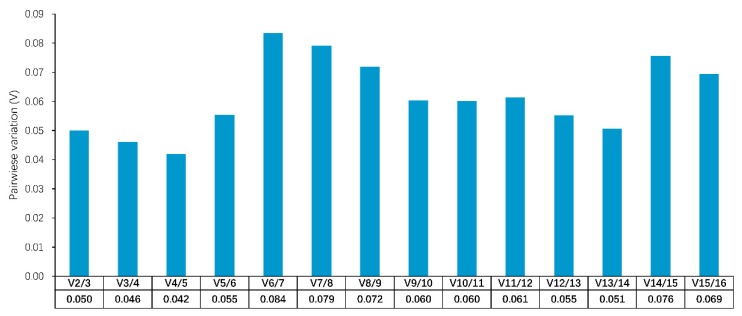
Pairwise variation (V) analyses in all tested samples. The V cutoff value was set to 0.15, below which the inclusion of an additional reference gene would not have a beneficial effect on the normalization.

The geNorm software (version 3.4) is a valid tool for identifying the optimal number of reference genes by calculating pairwise variation (V-value). If the V-value is less than 0.15, there is no need to add additional genes for normalization [23]. These results showed the V-value ranged from 0.042 to 0.084, which were all lower than the suggested threshold value of 0.15 (Figure 2). Therefore, two genes would be sufficient for accurate normalization.

### 3.5. Effects of Reference Gene Choice

**Figure 3:**
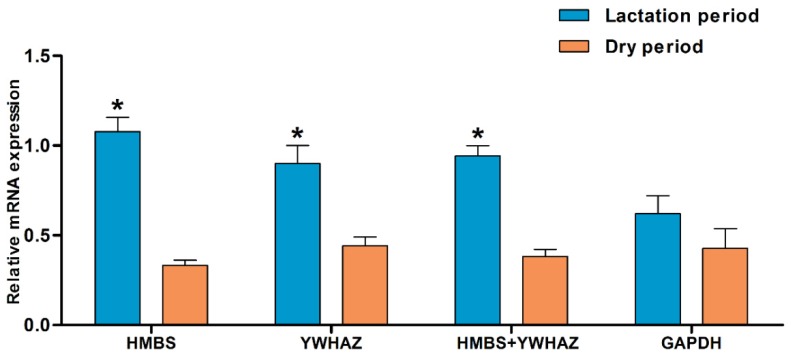
The normalized expression of the *RPL35* mRNA in yak mammary gland at lactation and dry period. The expression levels were normalized using HMBS, YWHAZ or GAPDH as reference genes individually and with the mean of HMBS+YWHAZ. Values are expressed as mean ± SE. * *p* < 0.05.

On the basis of the overall comprehensive ranking, we compared the expression profiles of *RPL35* gene in yak mammary gland using the two most stable genes (*HMBS* and *YWHAZ*) and the least stable gene (*GAPDH*) as reference genes. The results indicate that different reference genes have significant influences on calculated expression levels of target genes. When the most stable reference genes *HMBS* and *YWHAZ* were used as a reference, *RPL35* expression was significantly increased in the lactation period as compared with the dry period, however, there was no significant difference when *GAPDH* was applied for normalization (Figure 3).

## 4. Discussion

The mammary gland is a complex organ for the biosynthesis and secretion of milk, which provides essential nutrients for human and neonatal offspring. It undergoes a cycle of cell division, differentiation, dedifferentiation, and cell death during the transition from lactation to dry period [28,29]. The mammary gland in lactation consists of a branching network formed by epithelial cells. After cessation of milk removal, mammary gland involution occurs, as does dedifferentiation and apoptosis of mammary epithelial cells [6,30]. Related gene expression analysis by RT-qPCR is a reliable and widely employed method to unravel the molecular mechanism of mammary gland development and lactation, however, RT-qPCR data can be easily affected by the expression stability of the reference genes chosen for gene expression normalization. The ideal reference gene should be continuously expressed in breeds, tissues, cells, and organisms throughout distinct development stages [15], however, no ideal reference gene exists. Thus, selection and validation of reference genes for data normalization in an experiment are recommended.

In this study, a comprehensive analysis of 16 candidate reference genes in lactation and dry periods of yak mammary gland was performed. Four commonly used statistical methods (geNorm, NormFinder, BestKeeper, and Delta Ct) were used to assess the stability of the candidate reference genes. The geNorm method assesses gene stability by computing pairwise comparisons and geometric averaging of each reference gene. Genes with values below 1.5 are considered excellent constitutive genes [23]. In contrast, the NormFinder method calculates stability value based on the estimates for both intra- and intergroup variations [24]. The BestKeeper method ranks the stable reference genes according to correlation coefficient as well as standard deviation (SD) and coefficient of variation (CV) calculation of Cq values [25]. The Delta Ct method is based on comparison of the variability of their Ct differences over the samples [26]. Due to different algorithms and analytical procedures, each statistical method produced a different stability ranking of reference genes. In our results, *YWHAZ* and *TBP* were the most stable reference genes across the samples using Delta Ct methods, which were similar to the NormFinder results. *HMBS* was recommended as the most stable gene using the geNorm and BestKeeper methods. To obtain consistent results, the RefFinder tool is widely used to generate a final comprehensive ranking of candidate reference genes [15]. In this study, the RefFinder tool suggested *HMBS* and *YWHAZ* as the most stable genes, whereas *GAPDH* was the least stable reference gene, which is consistent with previous results in cattle [31]. It has been reported that stability of reference genes was variable among species and breeds [32]. In some previous studies, *UXT*, *RPS9,* and *RPS15* were recommended as the most suitable reference genes in the bovine mammary gland during lactation [31]. Eukaryotic translation elongation factor 1 alpha 1 (*EEF1A1*), ribosomal protein L4 (*RPl4*), *B2M,* and ribosomal protein S15a (*RPS15A*) genes showed stability in bubaline mammary gland during different physiological stages [33]. Mitochondrial ribosomal protein S15 (*MRPS15*), ribosomal protein S23 (*RPS23*), and *UXT* genes were identified as the most stable reference genes in mammary tissue of lactating yak breed from Sichuan Province in China [34]. These results suggested the most stable reference genes are highly specific to the present experiment. If species, breed, and physiological periods of the sampled animals change in the experimental model, reference genes should be evaluated for each individual experiment.

HMBS belongs to the hydroxymethylbilane synthase superfamily, which serves an important role in the condensation process of four porphobilinogen molecules into the linear hydroxymethylbilane [35]. In previous studies, *HMBS* had a relatively constant expression in the muscular tissues of cattle, goat, and chicken [36,37,38] and in lung tissues of goat [39]. YWHAZ is a member of the 14-3-3 family of proteins which modulates signal transduction pathway via binding to phosphoserine-containing proteins. YWHAZ is related to a wide range of biological processes such as metabolism, protein trafficking, signal transduction, apoptosis, and cell cycle regulation [40,41]. Similar to our selected reference genes, *YWHAZ* was described as a stable transcribed gene in closely related species, for example, cattle, buffalo, and goat [42,43,44]. Use of multiple reference genes to calibrate the RT-qPCR data is an effective way to ensure the robustness of results [13]. In this study, the geNorm analysis showed that the pairwise variation value of V2/V3 was below 0.15 for the two top ranked reference genes, *HMBS* and *YWHAZ*, and, therefore, they are appropriate for gene expression normalization in the yak mammary gland during lactation and the dry period. The *GAPDH* gene has been used the most for gene expression normalization in various tissues of yak [45,46]. In this study, the results of five statistical methods demonstrated that *GAPDH* was the least stable reference gene, which suggested that it is not a suitable reference gene for the Ashidan yak.

To further validate the selected reference genes, the relative expression pattern of the *RPL35* gene was measured in mammary glands of the Ashidan yak. The *RPL35* gene encodes a ribosomal protein that is a component of the 60S subunit, which is involved in the Met-mediated regulation of CSN2 translational elongation and secretion [47]. When the two most stable genes (*HMBS* and *YWHAZ*) were used to normalize the data, expression patterns of the *RPL35* gene showed a significant increase during the lactation period. This expression pattern is consistent with the results in Holstein-Friesian cattle [48], which confirmed the most stable reference genes of our study. By contrast, there was no significant difference between lactation and dry periods when the most unstable gene (*GAPDH*) was used as a reference gene. This result indicated that using an unstable reference gene could generate an inaccurate evaluation of target gene expression.

## 5. Conclusions

In conclusion, we used five statistical methods to determine the expression stability of 16 candidate reference genes in the yak mammary gland during the lactation and dry period. On the basis of the gene stability analysis, we recommend *HMBS* and *YWHAZ* as the suitable reference genes for normalization of gene expression. Our results could be useful for improving the accuracy of gene expression analysis in mammary gland of the Ashidan yak, with the potential of discovering candidate genes and as a tool to improve milk yield and quality traits, for example, by identifying expression quantitative trait loci (eQTLs).

## Figures and Tables

**Table 1:** The primers and amplification characteristics of sixteen candidate reference genes and one target gene.

Gene	NCBI Accession No.	Primer Sequence (5’–3’)	Size (bp)	Amplification Efficiency (%)	R^2^
*ACTB*	XM_005887322.2	F: ATTGCCGATGGTGATGAC R: ACGGAGCGTGGCTACAG	177	90.0	0.99
*GAPDH*	XM_014482068.1	F: TCACCAGGGCTGCTTTTA R: CTGTGCCGTTGAACTTGC	126	105.0	1.00
*UXT*	XM_005899362.2	F: AGGTGGATTTGGGCTGTAAC R: CTTGGTGAGGTTGTCGCTGA	170	105.0	1.00
*TBP*	XM_005908677.2	F: GTCCAATGATGCCTTACGG R: TGCTGCTCCTCCAGAATAGA	82	94.0	0.99
*YWHAZ*	XM_005887010.2	F: AATGTTGTAGGAGCCCGTAG R: CTGCTTGTGAAGCGTTGG	190	91.0	1.00
*RPL13A*	XM_014481217.1	F: CAAGCGGATGAACACCAA R: GCAGCAGGAACCACCATT	192	91.0	1.00
*SDHA*	XM_005894659.2	F: GGGAACATGGAGGAGGACA R: CCAAAGGCACGCTGGTAGA	188	106.0	0.99
*RPS15*	XM_005890466.2	F: GACCTTCCGCAAGTTCACCT R: ACCACCTCGGGCTTCTCCAT	198	101.0	1.00
*HPRT1*	XM_005911180.2	F: GTGATGAAGGAGATGGG R: ACAGGTCGGCAAAGAAC	79	108.0	0.99
*PPIA*	XM_005891872.2	F: TTTTGAAGCATACAGGTCC R: CCACTCAGTCTTGGCAGT	98	93.0	0.99
*HMBS*	XM_005897126.2	F: GAACAAAGGAGCCAAGAAC R: CAGAGGGCTGGGATGTAG	121	101.0	1.00
*MRPL39*	XM_005898618.2	F: AAACCTTTGACCAAGTCCTGT R: TTCCTCTTTGAATGCCCTCTC	135	94.0	0.99
*PPP1R11*	XM_005911410.2	F: CAGAAAAGACAGAAGGGTGC R: TTCCGAAGTTTGATGGTTAG	164	100.0	0.99
*B2M*	XM_005911364.2	F: CTGAGGAATGGGGAGAAG R: TGGGACAGCAGGTAGAAA	80	93.0	0.99
*RPS9*	XM_014483477.1	F: ACATCCCGTCCTTCATCGTGC R: GCCACTGCACCTTGTAACACT	123	1.06	0.99
*MTG1*	XM_005891439.2	F: GTGATGTCCAGGATTCAGGTGT R: AAGGAAGTCAGCCAGGGTCT	165	90.0	0.99
*RPL35*	XM_005893076.2	F: ATCCGAGTGGTTCGTAAATC R: GCTGCTGCTTCTTGGTCTTC	126	104.0	0.99

**Table 2:** Stability of reference genes in yak mammary gland at lactation and dry period.

Rank	GeNorm		NormFinder		BestKeeper		Delta Ct		ReFinedr	
1	*HMBS*	0.13	*YWHAZ*	0.22	*HMBS*	0.40	*YWHAZ*	0.61	*HMBS*	2.51
2	*UXT*	0.13	*TBP*	0.24	*RPS15*	0.41	*TBP*	0.65	*YWHAZ*	2.63
3	*PPIA*	0.15	*PPP1R11*	0.41	*UXT*	0.47	*PPIA*	0.68	*TBP*	3.74
4	*RPL13A*	0.18	*RPS15*	0.42	*RPS9*	0.48	*RPS15*	0.70	*UXT*	3.81
5	*HPRT1*	0.20	*ACTB*	0.46	*RPL13A*	0.52	*HMBS*	0.71	*RPS15*	4.00
6	*YWHAZ*	0.25	*PPIA*	0.48	*PPIA*	0.52	*PPP1R11*	0.72	*PPIA*	4.24
7	*TBP*	0.35	*RPS9*	0.48	*TBP*	0.52	*UXT*	0.72	*PPP1R11*	6.50
8	*RPS15*	0.43	*HMBS*	0.53	*YWHAZ*	0.57	*RPS9*	0.74	*RPS9*	6.70
9	*RPS9*	0.49	*MTG1*	0.56	*PPP1R11*	0.59	*HPRT1*	0.74	*RPL13A*	7.17
10	*B2M*	0.53	*UXT*	0.58	*HPRT1*	0.62	*ACTB*	0.74	*HPRT1*	8.39
11	*PPP1R11*	0.57	*HPRT1*	0.59	*MRPL39*	0.72	*RPL13A*	0.75	*ACTB*	9.40
12	*ACTB*	0.61	*RPL13A*	0.62	*B2M*	0.77	*MTG1*	0.80	*MTG1*	12.06
13	*MRPL39*	0.64	*MRPL39*	0.64	*ACTB*	0.80	*MRPL39*	0.85	*B2M*	12.38
14	*MTG1*	0.66	*B2M*	0.77	*MTG1*	0.88	*B2M*	0.92	*MRPL39*	12.47
15	*SDHA*	0.74	*SDHA*	1.05	*SDHA*	1.04	*SDHA*	1.15	*SDHA*	15.00
16	*GAPDH*	0.79	*GAPDH*	1.10	*GAPDH*	1.21	*GAPDH*	1.18	*GAPDH*	16.00

## Supplementary Materials

The following are available online at https://www.mdpi.com/2076-2615/9/11/943/s1, Figure S1: Melting curves of 16 candidate reference genes showing single peaks.
