# Supplementary figures and images for: The Selection of Reference Genes for Quantitative Real-Time PCR in the Ashidan Yak Mammary Gland During Lactation and Dry Period
Source: Animals (Basel). 2019 Nov 10;9(11):943. doi: 10.3390/ani9110943 (PMC6912359; doi:10.3390/ani9110943)

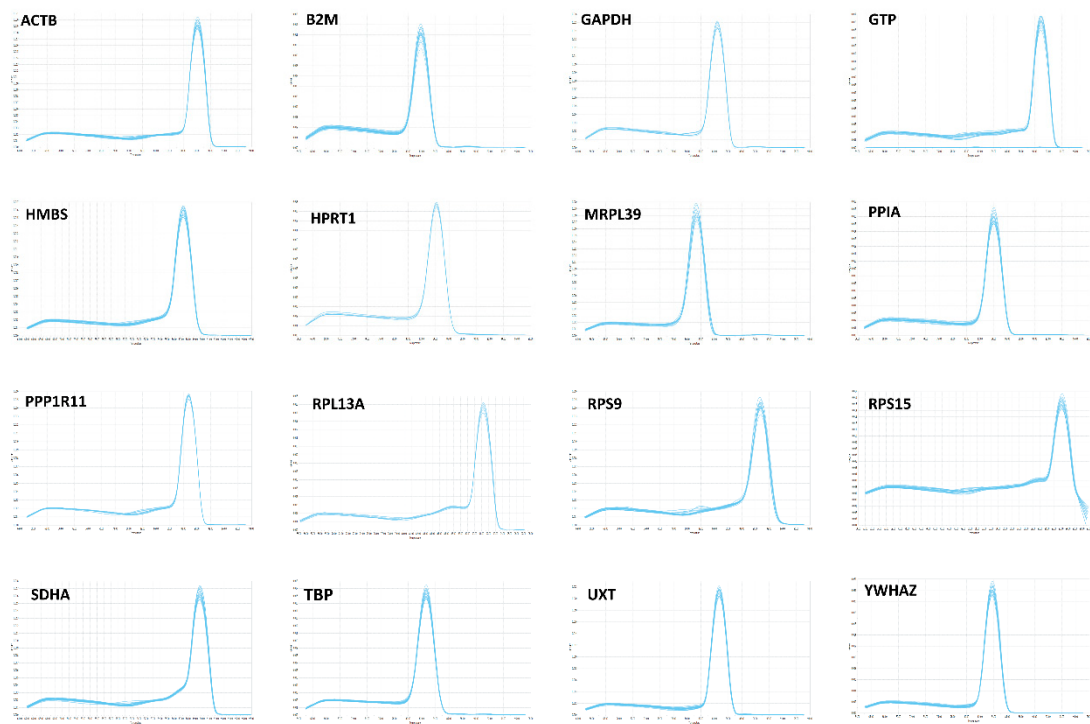

**Figure S1.** Melting curves of 16 candidate reference genes showing single peaks.

Supplement: Supplementary file 1 [file animals-09-00943-s001.pdf]
